# Inflammatory Biomarkers and Outcome Heterogeneity in Anti-MDA5 Antibody-Associated Interstitial Lung Disease: A Single-Center Consecutive Cohort Study

**DOI:** 10.3390/arm94030029

**Published:** 2026-04-28

**Authors:** Akina Nigi, Keisuke Iwamoto, Hidetoshi Itani, Shigeto Kondou, Yuki Okunishi, Takahiro Ohnishi

**Affiliations:** 1 Department of Respiratory Medicine, Japanese Red Cross Ise Hospital, 471-2 Funae, Ise 516-8512, Mie, Japan; momonga5170404@yahoo.co.jp (K.I.); ittann0630@gmail.com (H.I.); shigeto@msj.biglobe.ne.jp (S.K.); 2 Department of Rheumatology Medicine, Japanese Red Cross Ise Hospital, 471-2 Funae, Ise 516-8512, Mie, Japan; y.okunishi0418@gmail.com (Y.O.); otakamanntulip88@gmail.com (T.O.)

**Keywords:** anti-MDA5 antibody, interstitial lung disease, ferritin, C-reactive protein, lactate dehydrogenase, biomarkers, mortality, dermatomyositis

## Abstract

**Highlights:**

**What are the main findings?**

- Peak LDH, CRP, and ferritin levels were higher in non-survivors with anti-MDA5 antibody-associated ILD.
- PCA showed partial separation between survivors and non-survivors, but substantial overlap remained across biomarker profiles.

**What are the implications of the main findings?**

- Inflammatory biomarkers may support exploratory risk assessment in anti-MDA5 antibody-associated ILD.
- However, single biomarkers alone are insufficient for precise prognostication, and the findings require validation in larger cohorts.

**Abstract:**

Background: Anti-melanoma differentiation-associated gene 5 (anti-MDA5) antibody-positive interstitial lung disease (ILD) is associated with high mortality. While inflammatory markers have been linked to poor outcomes, clinical heterogeneity remains evident, as some patients survive despite marked hyperinflammation. Methods: We retrospectively analyzed consecutive patients with anti-MDA5 antibody-positive ILD treated at our institution between May 2017 and November 2025. In-hospital mortality was assessed in relation to clinical characteristics and laboratory markers, including peak anti-MDA5 antibody titers, ferritin, C-reactive protein (CRP), lactate dehydrogenase (LDH), and KL-6. Analyses were exploratory and hypothesis-generating. Continuous variables were compared using Mann–Whitney U tests, and categorical variables using Fisher’s exact test. Principal component analysis (PCA) and receiver operating characteristic (ROC) analyses were performed for descriptive purposes. Results: Seventeen patients were included (10 survivors and 7 non-survivors). Peak ferritin, C-reactive protein (CRP), and lactate dehydrogenase (LDH) levels were significantly higher in non-survivors, whereas peak anti-MDA5 antibody titers showed a non-significant trend toward higher values in non-survivors (*p* = 0.057). KL-6 levels did not differ significantly between groups. In ROC analyses, LDH and CRP showed the highest discriminative performance for in-hospital mortality, followed by ferritin, whereas KL-6 showed the lowest discriminative performance. Despite these overall trends, substantial overlap between survivors and non-survivors remained across all biomarkers. Principal component analysis (PCA) demonstrated partial separation of outcomes along an inflammation-dominant axis, but with persistent overlap, indicating marked outcome heterogeneity. Conclusions: Inflammatory biomarkers, particularly LDH, CRP, and ferritin, were associated with in-hospital mortality in anti-MDA5 antibody-associated ILD. However, persistent overlap between survivors and non-survivors suggests that single-biomarker assessment is insufficient for precise prognostication. These findings should be interpreted as hypothesis-generating and require validation in larger multicenter cohorts.

## 1. Introduction

Anti-melanoma differentiation-associated gene 5 (anti-MDA5) antibody-positive interstitial lung disease (ILD) is a severe clinical entity frequently associated with rapidly progressive respiratory failure and high short-term mortality. Despite advances in combination immunosuppressive strategies—including corticosteroids, calcineurin inhibitors, cyclophosphamide, and more recently Janus kinase (JAK) inhibitors—early mortality remains substantial in a subset of patients [1,2,3]. Early identification of individuals at high risk of deterioration remains a major clinical challenge.

Previous studies have identified several laboratory markers associated with poor prognosis in anti-MDA5-associated ILD. Elevated serum ferritin has consistently been reported as a marker of hyperinflammation and macrophage activation and has been linked to increased mortality [1,2,4]. C-reactive protein (CRP) and lactate dehydrogenase (LDH) have similarly been associated with disease severity and lung injury [2,5]. Krebs von den Lungen-6 (KL-6), a marker of alveolar epithelial injury, has also been evaluated as a potential indicator of pulmonary damage and prognosis [6]. These findings support the concept that systemic inflammation and immune dysregulation play central roles in disease progression.

In addition to inflammatory markers, anti-MDA5 antibody titers themselves have been proposed as a surrogate of disease activity. Some cohorts have suggested that higher antibody levels correlate with more severe lung involvement or worse outcomes [1], whereas others have reported inconsistent or limited prognostic utility for short-term mortality [7,8]. Thus, the independent contribution of antibody burden to acute outcome remains uncertain.

Importantly, substantial heterogeneity in clinical outcomes is observed in anti-MDA5 antibody-associated ILD. Some patients with markedly elevated inflammatory markers survive following intensive therapy, whereas others deteriorate rapidly despite apparently comparable laboratory profiles. Recent analyses employing multidimensional or cluster-based approaches have suggested the presence of biologically heterogeneous phenotypes within ILD populations [9], underscoring the limitations of relying solely on individual biomarkers for prognostication.

Clarifying the extent to which systemic inflammatory burden and antibody titers are associated with in-hospital mortality, and evaluating the degree of overlap between survivors and non-survivors, may provide a more nuanced understanding of risk stratification in this condition. Therefore, we aimed to explore the relationship between clinical characteristics, peak laboratory biomarkers, and in-hospital mortality in patients with anti-MDA5 antibody-positive ILD, with particular emphasis on outcome heterogeneity and the practical limitations of single-marker risk prediction.

## 2. Materials and Methods

### 2.1. Study Design and Patients

This retrospective, single-center study included consecutive patients with anti-melanoma differentiation-associated anti-MDA5 antibody-positive interstitial lung disease (ILD) treated at our institution between May 2017 and November 2025. Anti-MDA5 antibody positivity was defined based on commercially available immunoassays performed as part of routine clinical care. All eligible patients diagnosed during the study period were included to minimize selection bias.

### 2.2. Clinical Data Collection

Clinical data were extracted from electronic medical records. Baseline characteristics included age, sex, clinical presentation, the presence of rapidly progressive ILD, and chest high-resolution computed tomography (HRCT) patterns at presentation. Baseline HRCT findings were classified descriptively according to the predominant radiologic pattern.

Laboratory variables of interest were selected a priori based on previous literature and clinical relevance, including peak anti-MDA5 antibody titers, ferritin, C-reactive protein (CRP), lactate dehydrogenase (LDH), and Krebs von den Lungen-6 (KL-6). Peak values during hospitalization were used for analysis to reflect maximal inflammatory burden. Oxygen requirement at clinical deterioration was recorded as a marker of respiratory severity.

Treatment-related variables, including systemic corticosteroids, calcineurin inhibitors, intravenous cyclophosphamide, plasma exchange, and tofacitinib, were collected descriptively. Due to the observational design and limited sample size, treatment effects were not formally compared between outcome groups.

### 2.3. Outcome Definition

The primary outcome was in-hospital mortality, defined as death from any cause during the index hospitalization. Patients discharged alive were classified as survivors. Length of hospital stay and follow-up duration were recorded descriptively but were not treated as independent outcome predictors, as they are inherently influenced by survival status.

### 2.4. Statistical Analysis

All analyses were exploratory and hypothesis-generating. Continuous variables were summarized as medians with interquartile ranges (IQRs) and compared between survivors and non-survivors using the Mann–Whitney U test. Categorical variables were expressed as counts and percentages and compared using Fisher’s exact test. No multivariable analyses were performed given the small sample size.

To explore patterns among multiple laboratory markers, principal component analysis (PCA) was conducted using standardized values of selected inflammatory markers. PCA was used descriptively to visualize relationships between variables and outcomes rather than for formal classification. PCA loadings for PC1 and PC2 were calculated from the standardized five-biomarker matrix and are shown in Supplementary Tables S1 and S2. Receiver operating characteristic (ROC) curve analyses were performed to assess the discriminative ability of selected biomarkers for in-hospital mortality, with results interpreted cautiously and without predefined cut-off values.

All statistical tests were performed using R (version 4.5.0) and Python (version 3.11). Bootstrap resampling (*n* = 2000; seed 12345) was used for ROC-related estimates. A two-sided *p* value < 0.05 was considered statistically significant.

## 3. Results

### 3.1. Patient Characteristics

A total of 17 patients with anti-MDA5 antibody-positive interstitial lung disease were included in this analysis. Of these, 10 patients survived to discharge, while 7 died during hospitalization, corresponding to an in-hospital mortality rate of 41.2%. Most patients in this cohort had rapidly progressive ILD. Baseline HRCT findings were predominantly characterized by organizing pneumonia (OP)-like patterns, although in some severe cases, radiologic progression toward diffuse alveolar damage (DAD)-like changes was observed during the clinical course. Baseline demographic characteristics and clinical features are summarized in Table 1. Baseline characteristics were compared descriptively. Non-survivors were older than survivors, whereas other baseline variables showed no clear differences in this small cohort.

### 3.2. Biomarker Distributions According to Clinical Outcome

**Figure 1:**
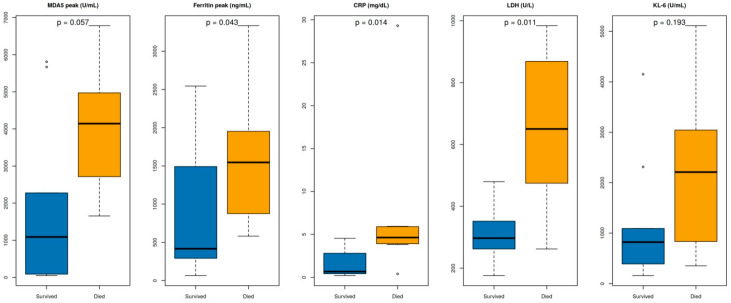
Distribution of peak biomarker levels according to outcome.

The distributions of peak serum biomarkers stratified by clinical outcome are shown in Figure 1. Peak anti-MDA5 antibody titers tended to be higher in non-survivors than in survivors; however, substantial overlap between groups was observed. Ferritin, CRP, and LDH levels were generally higher in non-survivors, whereas KL-6 showed substantial overlap between groups and did not significantly differ by outcome. Overall, no single biomarker completely separated survivors from non-survivors, underscoring marked heterogeneity in biomarker profiles among patients.

Figure 1 Boxplots of peak biomarker levels according to in-hospital outcome in patients with anti-MDA5 antibody-positive interstitial lung disease. Peak levels of anti-MDA5 antibody, ferritin, C-reactive protein (CRP), lactate dehydrogenase (LDH), and Krebs von den Lungen-6 (KL-6) are shown separately for survivors and non-survivors. Ferritin, CRP, and LDH were significantly higher in non-survivors, whereas anti-MDA5 antibody and KL-6 did not reach statistical significance. *p* values were calculated using the Mann–Whitney U test. Circles indicate outlier values.

### 3.3. Exploratory ROC Analysis

**Figure 2:**
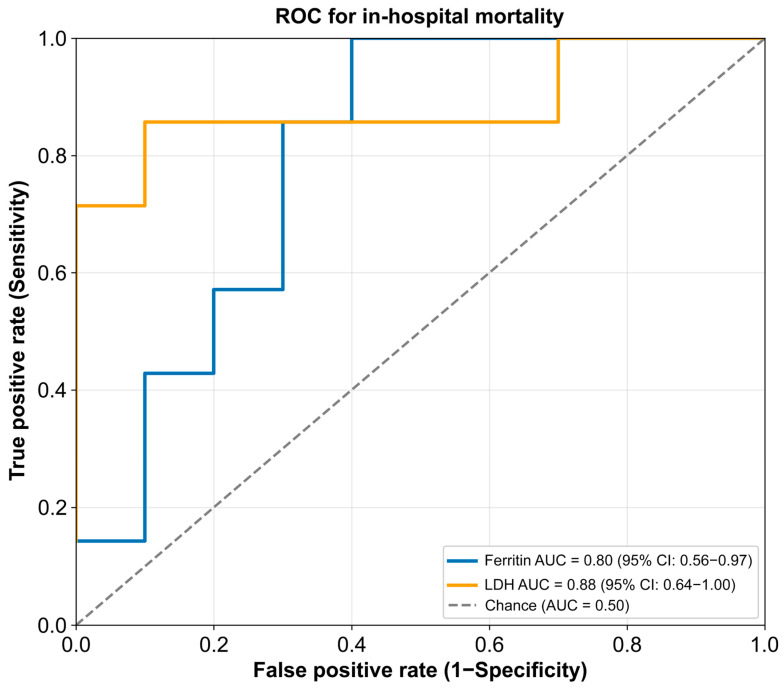
ROC curve for ferritin and LDH in predicting in-hospital mortality.

**Figure 3:**
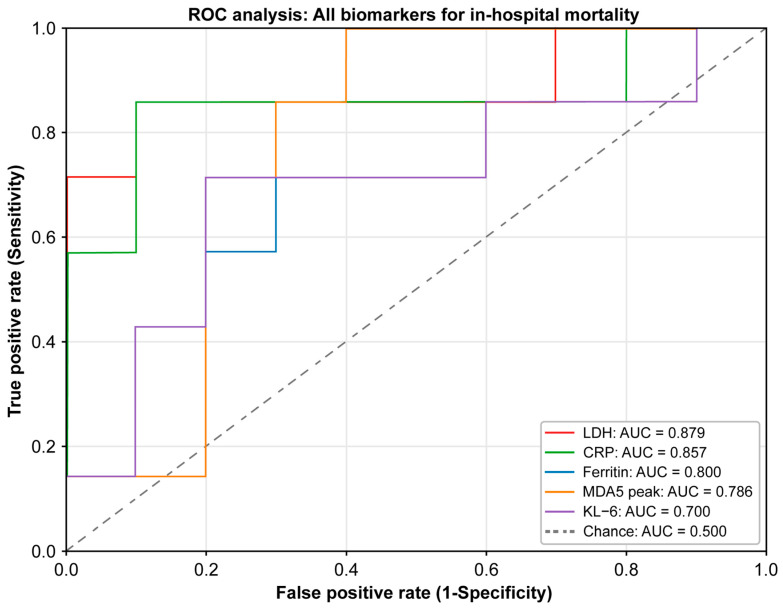
ROC curves of all evaluated biomarkers for predicting in-hospital mortality.

Exploratory receiver operating characteristic (ROC) analyses were performed to assess the discriminative ability of individual biomarkers for in-hospital mortality (Figure 2 and Figure 3). LDH showed the highest apparent discriminative performance [area under the curve (AUC) 0.879, 95% confidence interval (CI): 0.636–1.000], followed by CRP (AUC 0.857, 95% CI: 0.600–1.000) and ferritin (AUC 0.800, 95% CI: 0.557–0.971). Peak anti-MDA5 antibody titers showed borderline but non-significant discrimination (AUC 0.786, *p* = 0.057), whereas KL-6 had the lowest discriminative performance (AUC 0.700, *p* = 0.193). Given the exploratory nature of the study and the limited sample size, no optimal cut-off values were defined.

Figure 2 Receiver operating characteristic (ROC) curves for ferritin and lactate dehydrogenase (LDH) in predicting in-hospital mortality in patients with anti-melanoma differentiation-associated gene 5 (anti-MDA5) antibody-positive interstitial lung disease. LDH showed the highest apparent discriminative performance (AUC = 0.88, 95% CI: 0.64–1.00), followed by ferritin (AUC = 0.80, 95% CI: 0.56–0.97).

Figure 3 Receiver operating characteristic (ROC) curves of all evaluated biomarkers for predicting in-hospital mortality in patients with anti-MDA5 antibody-positive interstitial lung disease. LDH showed the highest apparent area under the curve (AUC = 0.879), followed by CRP (AUC = 0.857), ferritin (AUC = 0.800), anti-MDA5 antibody peak level (AUC = 0.786), and KL-6 (AUC = 0.700). The dashed diagonal line indicates chance performance.

### 3.4. Principal Component Analysis

**Figure 4:**
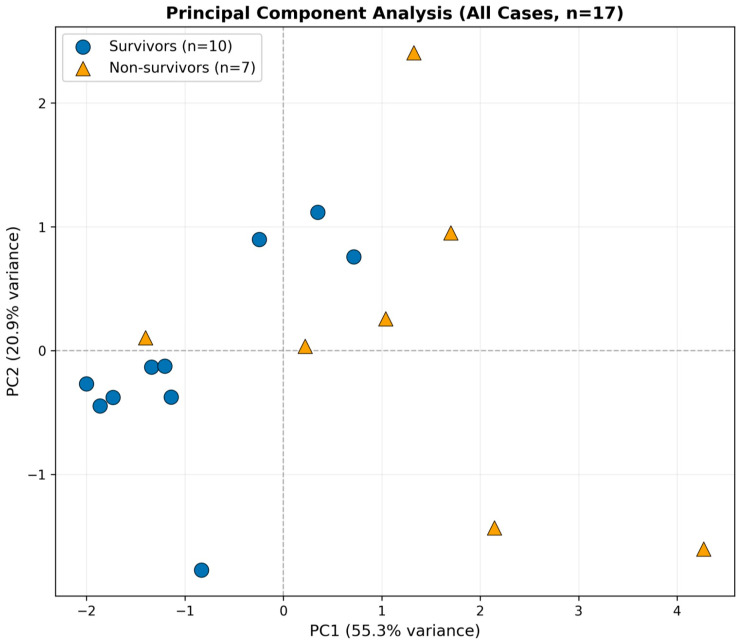
Principal component analysis of all cases. Dashed lines indicate zero lines of PC1 and PC2.

**Figure 5:**
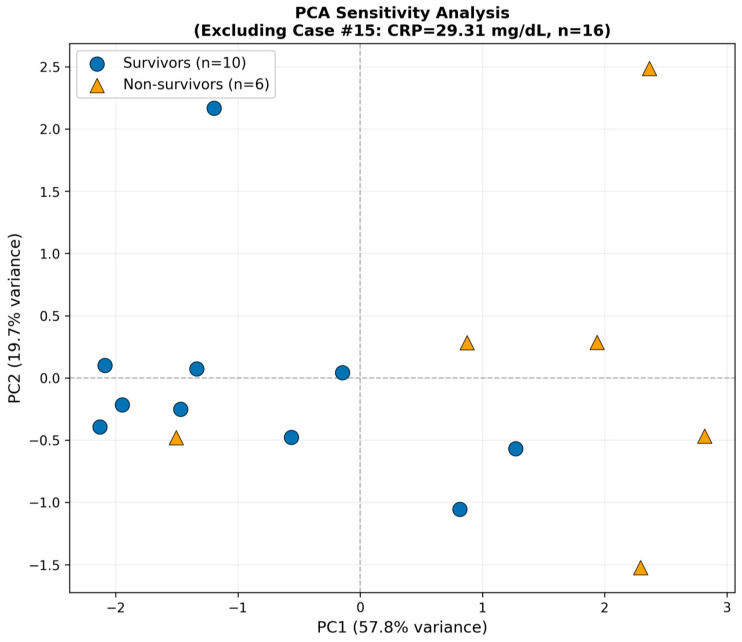
Sensitivity analysis of principal component analysis excluding the CRP outlier. Dashed lines indicate zero lines of PC1 and PC2.

Principal component analysis (PCA) was conducted as an exploratory visualization to examine multivariate relationships among biomarkers, including anti-MDA5 antibody titers, ferritin, KL-6, CRP, and LDH (Figure 4). Survivors and non-survivors showed partial separation along the first principal component (PC1), which explained 55.3% of the total variance, whereas the second principal component (PC2) explained 20.9%. Loading analysis showed that all five biomarkers loaded positively on PC1, with the largest weights for LDH, MDA5 peak, and CRP. In contrast, PC2 was driven mainly by ferritin and KL-6, suggesting a secondary and partially distinct biomarker pattern. However, substantial overlap remained between groups, indicating incomplete discrimination based on combined biomarker patterns. Because PCA is sensitive to extreme values, an additional analysis excluding one case with an extremely high CRP value (29.31 mg/dL) was performed for visualization purposes only (Figure 5). After re-standardization and re-analysis of the remaining 16 cases, the overall pattern of partial overlap persisted (PC1 57.8%, PC2 19.7%), suggesting that the PCA pattern was not solely driven by the outlying CRP value. PCA loading values for PC1 and PC2 were also examined to aid interpretation of the component structure, and these are provided in Supplementary Tables S1 and S2.

Figure 4 Principal component analysis (PCA) of all included cases (*n* = 17) based on biomarker profiles. Survivors (blue circles, *n* = 10) and non-survivors (orange triangles, *n* = 7) are displayed in the space defined by the first two principal components. PC1 explained 55.3% of the variance and PC2 explained 20.9%. A tendency toward separation between survivors and non-survivors was observed along PC1, although substantial overlap remained.

Figure 5 Sensitivity analysis of principal component analysis (PCA) after excluding Case #15, which showed the highest CRP value (29.31 mg/dL). Survivors (blue circles, *n* = 10) and non-survivors (orange triangles, *n* = 6) are shown in the space defined by the first two principal components. PC1 explained 57.8% of the variance and PC2 explained 19.7%. The overall tendency toward separation along the inflammatory biomarker axis was preserved, suggesting that the PCA pattern was not driven solely by a single outlier. In the sensitivity analysis, the overall PC1 structure was preserved, whereas PC2 became more strongly influenced by KL-6, indicating that the secondary component was somewhat more KL-6-dominant after exclusion of the extreme CRP outlier.

### 3.5. Treatment Overview

Treatment strategies are summarized descriptively in Table 2. Most patients received combination immunosuppressive therapy, including systemic corticosteroids and calcineurin inhibitors. Intravenous cyclophosphamide was administered in a subset of patients. In addition, tofacitinib-based therapy was used in five patients as part of combination treatment. Plasma exchange was performed in selected cases. Given the observational design and limited sample size, no formal comparisons of treatment efficacy between survivors and non-survivors were performed.

## 4. Discussion

Anti-melanoma differentiation-associated gene 5 (anti-MDA5) antibody-positive interstitial lung disease (ILD) remains a life-threatening condition despite advances in aggressive immunosuppressive therapy. Rapidly progressive forms are characterized by acute respiratory deterioration, and several cohorts have reported mortality rates approaching 30–50% [1,2,3,4]. Current treatment strategies typically involve early combination therapy with corticosteroids, calcineurin inhibitors, and cyclophosphamide [5], and more recently Janus kinase (JAK) inhibitors have also been incorporated into therapeutic regimens [6,7]. Plasma exchange has additionally been used in selected patients. Although JAK inhibitors and plasma exchange have emerged as promising options, their optimal indications remain under debate. Treatment responsiveness may vary according to factors such as age, treatment intensity, and the underlying immune status of individual patients.

From a pathophysiological perspective, anti-MDA5-associated ILD is a multifaceted condition that cannot be explained by a single inflammatory pathway. Both humoral and cellular immune mechanisms appear to contribute to disease progression. Autoantibody production reflects abnormalities in humoral immunity, whereas activation of cellular immune pathways—including macrophage activation and type I interferon-mediated responses—has been suggested to play a central role in disease pathogenesis [8,9,10,11,12,13]. Hyperferritinemia is often interpreted as a surrogate marker of macrophage activation and systemic hyperinflammation, while elevations in CRP and LDH may reflect downstream tissue injury and amplification of inflammatory responses [14]. In some patients, these processes may evolve into a cytokine storm-like state, leading to rapid alveolar damage and acute respiratory failure. In others, immune responses may progress in a more heterogeneous or temporally variable manner. In addition, patient-related factors—including baseline immune responsiveness, rate of disease progression, stage at presentation, and treatment intensity—may contribute to variability in clinical outcomes. Taken together, these biological and clinical differences suggest that anti-MDA5-associated ILD may represent a spectrum of immune-mediated lung injury rather than a uniform inflammatory disorder.

Given this complexity and heterogeneity in treatment response, it is difficult for a single biomarker to fully capture disease behavior or prognosis in anti-MDA5-associated ILD. Although inflammatory markers have repeatedly been reported to correlate with poor outcomes, the actual clinical course is likely shaped by interactions among multiple immune pathways and clinical factors rather than isolated elevations of individual laboratory parameters.

In this exploratory single-center study, we examined the relationship between peak inflammatory biomarker levels and in-hospital mortality. Consistent with previous reports, ferritin, CRP, and LDH were significantly higher in non-survivors. Despite the small cohort size, the presence of statistically significant differences between outcome groups for LDH, CRP, and ferritin suggests that systemic inflammatory burden may be relatively strongly associated with in-hospital mortality in this condition.

However, substantial overlap between survivors and non-survivors was observed across all evaluated biomarkers, as shown in Figure 1. Some patients with markedly elevated ferritin, CRP, or LDH survived following intensive therapy, whereas others with relatively modest laboratory abnormalities experienced fatal outcomes. These findings suggest that although inflammatory burden contributes to prognosis, individual biomarkers alone are insufficient to deterministically define clinical outcomes. Treatment-related heterogeneity may also have contributed to the observed overlap between biomarker levels and outcomes. Some patients with marked inflammatory abnormalities may have survived because of earlier or more intensive treatment, suggesting that therapeutic intervention may have modified the relationship between biomarker burden and mortality. Nevertheless, in a cohort of this size, such interpretations should remain cautious and hypothesis-generating.

ROC analyses showed that LDH and CRP demonstrated the highest apparent discrimination for in-hospital mortality (Figure 2 and Figure 3), with ferritin also providing a meaningful signal. Nevertheless, confidence intervals remained wide and overlap between groups persisted. These findings indicate that although inflammatory markers may provide supportive information for risk assessment in small real-world cohorts, they are insufficient for deterministic clinical decision-making when used in isolation. Overall, our results suggest that inflammatory burden may increase the probability of mortality but does not define a clear boundary separating survival from death.

In our cohort, peak anti-MDA5 antibody titers tended to be higher in non-survivors, although the difference did not reach statistical significance. The prognostic significance of antibody titers remains controversial. Some studies have suggested correlations with disease activity or severity [15,16,17], whereas others have reported limited predictive value for short-term mortality [10,11]. Our findings are consistent with the interpretation that antibody positivity may contribute to defining the disease phenotype but may not independently determine acute clinical outcomes. Antibody burden alone may not adequately capture the dynamic immunologic processes driving lung injury, which evolve over time and interact with host immune regulation. For example, plasma exchange can rapidly reduce circulating antibody levels, yet antibody titers may not necessarily reflect the current inflammatory activity of the disease. It is therefore possible that antibody levels reflect more chronic aspects of disease biology, whereas acute disease progression may involve additional immune mechanisms that change over shorter timescales.

Principal component analysis yielded similar findings (Figure 4 and Figure 5). Along the inflammatory biomarker axis (PC1), a tendency toward separation between survivors and non-survivors was observed; however, some overlap remained. Importantly, this overall pattern was preserved in sensitivity analyses, indicating that the findings were not driven solely by a small number of outliers.

Therefore, the clinical heterogeneity observed in anti-MDA5-associated ILD may not be explained solely by quantitative differences in individual biomarker levels but may reflect variations in immune regulation, disease progression dynamics, and stage at presentation. The radiologic features in our cohort also support this dynamic interpretation. Most patients had rapidly progressive ILD, and baseline HRCT findings were often OP-like; however, some severe cases showed radiologic progression toward DAD-like changes over time. This pattern suggests that a single baseline imaging category may not fully capture subsequent disease behavior in anti-MDA5 antibody-associated ILD. These observations are consistent with previous reports suggesting the presence of biologically heterogeneous phenotypes within ILD populations [18].

The diversity of treatment strategies used in clinical practice may itself reflect the immunologic complexity of this condition. In real-world settings, combinations of corticosteroids, calcineurin inhibitors, cyclophosphamide, plasma exchange, and JAK inhibitors are frequently employed [19,20,21,22]. Such therapeutic heterogeneity may indicate that different immune axes predominate in different patients. For example, interferon-driven pathways may play a dominant role in some individuals, whereas macrophage-driven hyperinflammation may be more prominent in others. These differences may partly explain why treatment responses vary even when aggressive combination therapy is administered.

Several limitations should be acknowledged. The retrospective design and limited sample size reduce statistical power and precluded multivariable adjustment. Age differed between outcome groups, and residual confounding cannot be excluded. Biomarker analyses were based on peak values during hospitalization, which may reflect treatment effects in addition to underlying disease severity. Furthermore, the single-center design limits generalizability, and the absence of an external validation cohort represents an important limitation in interpreting these findings.

Despite these limitations, our findings provide a practical illustration of real-world heterogeneity in anti-MDA5-associated ILD. Systemic inflammation was closely associated with short-term mortality, yet the persistent overlap of biomarker profiles highlights the limitations of deterministic risk prediction based on single laboratory markers. More refined stratification strategies may require integration of clinical parameters, dynamic immune profiling, and temporal assessment of disease activity rather than reliance on static biomarker thresholds alone.

## 5. Conclusions

In this small single-center cohort, elevated inflammatory biomarkers, particularly LDH, CRP, and ferritin, appeared to be associated with in-hospital mortality in anti-MDA5 antibody-associated interstitial lung disease. These findings suggest a possible role for these markers in exploratory risk assessment, while underscoring the substantial outcome heterogeneity that warrants validation in larger multicenter studies.

## Figures and Tables

**Table 1:** Patient Characteristics and Biomarker Performance.

Variable	All Patients (*n* = 17)	Survivors (*n* = 10)	Non-Survivors (*n* = 7)	AUC	95% CI	*p*-Value	Effect Size
Age (years)	66 [50–78]	52 [45.5–65.0]	75 [70.5–81.5]	-	-	0.017 *	0.714
Sex—Female, *n* (%)	13 (76.5%)	8 (80.0%)	5 (71.4%)	-	-	-	
Oxygen requirement, *n* (%)	10 (58.8%)	4 (40.0%)	6 (85.7%)	-	-	0.134	
MDA5 peak (U/mL)	2070 [1000–4295]	1090 [101.75–2152.5]	4145 [2715–4972.5]	0.786	0.529–1.000	0.057	0.571
Ferritin peak (ng/mL)	819 [414–1563]	416 [298.25–1287]	1546 [875–1952]	0.800	0.557–0.971	0.043 *	0.600
CRP (mg/dL)	2.80 [0.50–4.54]	0.665 [0.44–2.3125]	4.63 [3.905–5.905]	0.857	0.600–1.000	0.014 *	0.714
LDH (U/L)	352 [271–482]	297 [264.25–343.25]	650 [474.5–869]	0.879	0.636–1.000	0.011 *	0.757
KL-6 (U/mL)	1046 [454–2314]	821 [405.25–1081.25]	2211 [834–3043.5]	0.700	0.414–0.943	0.193	0.400

Continuous variables are presented as median [interquartile range]. Categorical variables are presented as *n* (%). AUC: area under the receiver operating characteristic curve. CI: confidence interval. Continuous variables were compared using the Mann–Whitney U test, and categorical variables using Fisher’s exact test. Effect sizes for continuous variables are presented as rank–biserial correlations, with positive values indicating higher levels in non-survivors. * *p* < 0.05.

**Table 2:** Treatment characteristics according to outcome.

Treatment	All Patients (*n* = 17)	Survivors (*n* = 10)	Non-Survivors (*n* = 7)
Systemic corticosteroids	16 (94.1%)	9 (90.0%)	7 (100%)
Calcineurin inhibitor (TAC/CyA)	13 (76.5%)	7 (70.0%)	6 (85.7%)
Cyclophosphamide (IVCY)	11 (64.7%)	6 (60.0%)	5 (71.4%)
Plasma exchange (PE)	6 (35.3%)	4 (40.0%)	2 (28.6%)
Tofacitinib	5 (29.4%)	3 (30.0%)	2 (28.6%)
Mechanical ventilation	0 (0%)	0 (0%)	0 (0%)

Values are presented as *n* (%).

## Data Availability

The original contributions presented in this study are included in the article/Supplementary Material. Further inquiries can be directed to the corresponding author.

## Supplementary Materials

The following supporting information can be downloaded at: https://www.mdpi.com/article/10.3390/arm94030029/s1, Supplementary Tables S1 and S2. Table S1:PCA loadings for PC1 and PC2 using all cases; Table S2: PCA loading for PC1 and PC2 after excluding the CRP outlier.

## Abbreviations

The following abbreviations are used in this manuscript:

anti-MDA5	anti-melanoma differentiation-associated gene 5
AUC	area under the curve
CRP	C-reactive protein
ILD	interstitial lung disease
IQR	interquartile range
KL-6	Krebs von den Lungen-6
LDH	lactate dehydrogenase
PCA	principal component analysis
ROC	receiver operating characteristic

## References

[1] Gono T., Kawaguchi Y., Kuwana M., Sugiura T., Furuya T., Takagi K., Ichida H., Katsumata Y., Hanaoka M., Ota Y. (2012). Anti-MDA5 antibody as a prognostic marker in dermatomyositis-associated interstitial lung disease. Arthritis Rheumatol..

[2] Tsuji H., Nakashima R., Hosono Y., Imura Y., Yagita M., Yoshifuji H., Hirata S., Nojima T., Sugiyama E., Hatta K. (2020). Multicenter prospective study of combined immunosuppressive therapy in anti-MDA5-positive dermatomyositis with interstitial lung disease. Rheumatology.

[3] Zhang S., Wu Q., Chen S. (2020). Predictors of prognosis in anti-MDA5 antibody-positive rapidly progressive interstitial lung disease. J. Rheumatol..

[4] Chen Z., Wang X., Ye S. (2020). Clinical and prognostic features of anti-MDA5 antibody-positive dermatomyositis-associated ILD. Rheumatol. Int..

[5] Cohen S.B., Kremer J.M., Dandreo K.J., Reed G.W., Magner R., Shan Y., Kafka S., DeHoratius R.J., Ellis L., Parenti D. (2019). Tacrolimus combined with glucocorticoids in anti-MDA5 dermatomyositis-associated ILD. Clin. Rheumatol..

[6] Kurasawa K., Arai S., Namiki Y., Tanaka A., Takamura Y., Owada T., Arima M., Maezawa R. (2018). Tofacitinib for refractory dermatomyositis-associated ILD. Mod. Rheumatol..

[7] Ishii T., Nakashima R., Hosono Y. (2022). Tofacitinib as rescue therapy for anti-MDA5-positive dermatomyositis-associated ILD. Rheumatology.

[8] Shu X., Ji J., Chen Z. (2023). Biomarkers in anti-MDA5-positive dermatomyositis-associated interstitial lung disease: A review. Front. Med..

[9] Yagishita M., Tsuboi H., Tabuchi D., Sugita T., Nishiyama T., Okamoto S., Terasaki T., Shimizu M., Honda F., Ohyama A. (2021). Prognostic factors in anti-MDA5 antibody-positive dermatomyositis-associated ILD. Mod. Rheumatol..

[10] Sugihara T., Kawahito Y., Morinobu A., Kaneko Y., Seto Y., Kojima T., Ito H., Kohno M., Nakayama T., Sobue Y. (2022). Predictors of treatment response in anti-MDA5-positive rapidly progressive ILD. Mod. Rheumatol..

[11] Abidi S.H., Almansour N.M., Amerzhanov D., Allemailem K.S., Rafaqat W., Ibrahim M.A.A., la Fleur P., Lukac M., Ali S. (2021). Predictive value of serum ferritin level and treatment response in anti-MDA5 antibody-positive interstitial lung disease. Sci. Rep..

[12] Greenberg S.A. (2010). Type I interferons and myositis. Arthritis Res.Ther..

[13] Gono T., Kawaguchi Y., Ozeki E., Ota Y., Satoh T., Kuwana M., Hara M., Yamanaka H. (2011). Serum ferritin correlates with activity of anti-MDA5 antibody-associated interstitial lung disease. Arthritis Rheumatol..

[14] Enteshari-Moghaddam A., Azami A., Isazadehfar K., Mohebbi H., Habibzadeh A., Jahanpanah P. (2019). Clinical significance of CRP levels in anti-MDA5-positive dermatomyositis-associated ILD. Clin. Rheumatol..

[15] Yamazaki S., Shimizu M., Akutsu Y., Shimbo A., Mori M. (2022). Real-world use of tofacitinib in dermatomyositis-associated ILD. Mod. Rheumatol..

[16] Wang Y., Jia W., Ma Q., Li F., Ma Z., Yang M., Pu J., Huo Z., Dang J. (2023). JAK inhibitor combined with plasma exchange improves survival in anti-MDA5-positive dermatomyositis. Clin. Exp. Rheumatol..

[17] López-de-Andrés A., Jiménez-García R., Esteban-Vasallo M.D., Hernández-Barrera V., Aragon-Sánchez J., Jiménez-Trujillo I., de Miguel-Diez J., Palomar-Gallego M.A., Romero-Maroto M., Perez-Farinos N. (2019). Plasma exchange and rituximab in anti-MDA5-positive rapidly progressive ILD. J. Clin. Med..

[18] Fang P., Liang J., Jiang X., Fang X., Wu M., Wei X., Yang W., Hou W., Zhang Q. (2020). Janus kinase inhibitors in refractory myositis: A real-world experience. Front. Pharmacol..

[19] Wu Q., Wang Y., Zhang S. (2022). Tofacitinib in rapidly progressive ILD associated with anti-MDA5 dermatomyositis. Front. Immunol..

[20] Liu Y., Liang Z., Yuan S., Wang S., Guo F., Peng W., Yang J., Wu A. (2022). Identification of clinical phenotypes in interstitial lung disease using cluster analysis. Respir. Res..

[21] He S., Zhen X., Hu Y. (2021). Plasma exchange in anti-MDA5 antibody-positive dermatomyositis with rapidly progressive ILD. Clin. Rheumatol..

[22] Ikumi K., Kobayashi S., Tamura N., Tada K., Inoue H., Osaga S., Nishida E., Morita A. (2019). Combination immunosuppressive therapy with plasma exchange in anti-MDA5 dermatomyositis-associated ILD. Mod. Rheumatol..

