# Supplementary material for: Inflammatory Biomarkers and Outcome Heterogeneity in Anti-MDA5 Antibody-Associated Interstitial Lung Disease: A Single-Center Consecutive Cohort Study
Source: Adv Respir Med. 2026 Apr 28;94(3):29. doi: 10.3390/arm94030029 (PMC13214490; doi:10.3390/arm94030029)
Supplement: Supplementary file 1 [file arm-94-00029-s001.zip › arm-4225669-supplementary.pdf]

Supplementary Table1. PCA loading for the five standardized biomarkers

Biomarker	PC1	PC2
MDA5 peak	0.513	0.152
Ferritin peak	0.303	0.749
CRP	0.479	-0.267
LDH	0.554	0.030
KL-6	0.330	-0.586

Supplementary Table2. PCA loading in the sensitivity analysis excluding Case#15

Biomarker	PC1	PC2
MDA5 peak	0.465	-0.083
Ferritin peak	0.445	-0.371
CRP	0.513	-0.066
LDH	0.530	0.091
KL-6	0.206	0.918
